# Nanomechanical characterization of nanostructured bainitic steel: Peak Force Microscopy and Nanoindentation with AFM

**DOI:** 10.1038/srep17164

**Published:** 2015-11-25

**Authors:** Lucia Morales-Rivas, Alejandro González-Orive, Carlos Garcia-Mateo, Alberto Hernández-Creus, Francisca G. Caballero, Luis Vázquez

**Affiliations:** 1 Department of Physical Metallurgy, National Center for Metallurgical Research (CENIM-CSIC), Avda. Gregorio del Amo 8, 28040 Madrid, Spain; 2 Departamento de Química Física, Facultad de Química, Universidad de La Laguna, 38206 Tenerife, Spain; 3 Instituto de Ciencia de Materiales de Madrid, (ICMM-CSIC), Cantoblanco, 28049 Madrid, Spain

## Abstract

The full understanding of the deformation mechanisms in nanostructured bainite requires the local characterization of its mechanical properties, which are expected to change from one phase, bainitic ferrite, to another, austenite. This study becomes a challenging process due to the bainitic nanostructured nature and high Young’s modulus. In this work, we have carried out such study by means of the combination of AFM-based techniques, such as nanoindentation and Peak Force Quantitative Nanomechanical Mapping (PF-QNM) measurements. We have addressed critically the limits and advantages of these techniques and been able to measure some elastoplastic parameters of both phases. Specifically, we have analyzed by PF-QNM two nanostructured bainitic steels, with a finer and a coarser structure, and found that both phases have a similar Young’s modulus.

Nanostructured bainite belongs to a new generation of advanced steels with improved mechanical properties, presenting the highest strength/toughness combinations ever recorded in bainitic steels (2.5 GPa/30 MPa m^1/2^)^1^,^2^,^3^,^4^. The difficulty to analyze the flow behavior of nanostructured bainite arises from the complexity of its structure and from the combination of different deformation-strengthening mechanisms, including the mechanically induced transformation of austenite into martensite. Under stress, nanostructured bainite responds as a composite-like structure with the stress and strain partitioning between the phases bainitic ferrite and retained austenite, as reported for other multiphase steels^5^,^6^,^7^.

The determination of local elastic properties is a previous and crucial step to be able to understand how mechanical partitioning occurs and thus, how this can affect the macroscopic flow behavior of the material. In fact, when a multiphase material is subjected to a macroscopic mechanical load, at the elastic regime, the phase with a higher Young’s modulus would undertake most of the stress, exerting thus a shielding effect over the phase with a lower E. This composite-type behavior influences the point at which yielding starts and the way it takes place, i.e., which phase reaches first its plastic regime. Moreover, elastic properties are an important factor influencing the development of residual stresses formed as a consequence of interactions among others such as time, temperature, deformation and microstructure^8^.

Elastic properties are expected to differ from one phase to another and both from the corresponding macroscopic value obtained from a mechanical test. Since their phases are not possible to be processed as mono-phase materials, the elastic properties of each phase must be individually measured. Therefore, traditional bulk measurements of elastic properties based on ultrasonic resonance, which is not phase-sensitive, are not suitable for multiphase structures^9^. Instead, and specifically for the determination of Young’s modulus, two different experimental techniques are commonly employed:

1- *In-situ* diffraction tests, where the Young’s modulus can be estimated by considering, in the elastic regime, the evolution of the longitudinal lattice strain with the stress. This technique gives information of the average Young’s modulus for each phase and for a specific family of planes considered^10^. So, based on diffraction techniques, different Young’s modulus values are calculated from the average lattice spacing of a group of grains with plane normals [hkl] directed along the scattering vector, which includes all crystal orientations rotated around that particular [hkl]^11^. Therefore, Young’s modulus thus obtained reflects the anisotropic nature of the single crystal. On the other hand, there is also an averaging which originates from the large variety of mechanical boundary conditions of this particular group of grains, each surrounded by other grains of unknown orientation^9^. The fact that the stress undertaken by a given group of grains is not directly measured is a disadvantage of this method. This must be estimated assuming a particular stress partitioning model, such as Reuss model that implicitly assumes the macroscopic stress^10^.

2-Determination of local Young’s modulus has been intensively performed by means of nanoindentation. The analysis typically consists in the examination of the unloading curve, for which analytical solutions for different indenter geometries are used^12^,^13^,^14^,^15^,^16^,^17^,^18^,^19^. This method, however, does not account for the pile-up, sink-in, and tip-blunting effects, which change the value of contact area, a key parameter on which results are strongly dependent^20^. Moreover, when dealing with a nanocrystalline structure, as it is our case, the Young’s modulus measured through this procedure is likely to be an average of the bulk material, since the footprint generated can have a size similar and even higher than the microstructural features to be measured. In addition, in the case of nanostructured bainite, as a steel presenting Transformation Induced Plasticity (TRIP) effect, the indentation might induce the martensitic transformation of austenite, so the initial Young’s modulus value is expected to differ from the measured one due to this phase transition.

In principle, AFM-based techniques could be apposite to address the study of the nanobainitic local elastic properties at the nanoscale. AFM working at different modes such as force curve analysis or Peak Force Quantitative Nano-mechanical (PF-QNM) could be a proper technique to obtain the Young’s modulus values of these nanocrystalline structures due to its improved lateral resolution and capabilities to perform very shallow indentations (i.e. at the nanoscale). Moreover, in the PF-QNM mode, it would be possible to obtain simultaneously morphological and stiffness (i.e. Young’s modulus) images of the same area. Therefore, local spatial variations of Young’s modulus due to the heterogeneity of the microstructure could be observed.

The aim of this work is to determine the local elastic properties of nanoscale composite-like bainitic structures, using the combination of AFM-based techniques. In special, the suitability of PF-QNM to obtain the Young’s modulus distribution in this material has been explored. A particular emphasis has been paid to discern whether bainitic ferrite and retained austenite have similar or dissimilar Young’s modulus. For this purpose, two different samples, transformed into bainite at different temperatures, have been analyzed by PF-QNM. In addition, results have been compared to those obtained from the analysis of the loading force curve of single indentations performed by AFM. Results have been discussed in terms of the nature and scale of the microstructures and have provided new experimental evidence on the mechanical behavior of nanoscale composite-like bainitic structures.

## Material and Experimental Procedure

### Material

The chemical composition of the steel is Fe-1.0C–2.5Si–0.75Mn–0.12Ni-1.0Cr-0.03Mo-0.2Cu (wt. %). The heat was industrially manufactured via electric arc furnaces. Once solidified, the ingot was reheated at 1200 °C and hot rolled to a 35 mm bar, which was afterward slowly cooled down in a furnace to avoid cracking. After austenitization at 950 °C during 60 min and 15 min for HT250 and HT350, respectively, two isothermal transformation treatments were selected: 250 °C during 16 h (sample HT250), to achieve a nanoscale bainitic structure, and 350 °C during 480 min (sample HT350), to achieve a sub-micron bainitic structure. Both temperatures are in the bainitic range and times are long enough to ensure the end of the transformation.

For the AFM experiments the sample preparation of undeformed material consisted of a first stage of grounding, followed by polishing using 3 μm and 1 μm diamond paste and several cycles of etching using 2% Nital (2% nitric acid in ethanol) and polishing, in order to remove the deformed layer. Samples were finally polished using colloidal silica suspension. After that, two different surface finishing processes were applied per heat treatment: etching (HT250 and HT350 with etched surface) or cleaning (HT250 and HT350 with polished surface). Etching was carried out using 2% Nital. Upon the corrosive chemical etching with Nital, the etchant removes the top layer of the metal in a selective way, depending on the phase and the crystal orientation, as opposed to the so-called color etching, in which a thin film is built on the top of the metal^21^,^22^. In turn, cleaning, intended to remove rests of silica colloidal, was made using a solution of 96 ml water, 2 ml ammonia water (30%) and 2 ml hydrogen peroxide (3%), based on^23^,^24^.

TEM specimens were sliced from 3-mm-diameter rods of the heat-treated material, mechanically thinned to 0.06 mm, and then twin-jet electropolished to perforation using a mixture of 5% perchloric acid, 25% glycerol and 70% ethanol at 10 °C at 45 V. The samples were examined on a TEM JEOL 2010 transmission electron microscope operated at 200 keV.

Quantitative XRD analysis was used to determine the lattice parameter of ferrite and austenite in HT250 and HT350 samples. They were then step-scanned in a Brucker-Axs D8 X-ray diffractometer using unfiltered Co Kα radiation. X-ray diffraction measurements were carried out with a Bruker AXS D8 diffractometer equipped with a Co X-ray tube, Goebel mirror optics and a LynxEye 6. Linear Position Sensitive Detector for ultra-fast XRD measurements. This type of radiation is especially suited for iron-rich samples to avoid the strong fluorescence arising from copper radiation, and to produce high resolution data. A current of 30 mA and a voltage of 40 kV were employed as tube settings. Operational conditions were selected to obtain XRD profiles of sufficient quality: namely, optimal counting statistics, narrow peaks and detection of the small diffraction peaks of minor phases. The XRD data were collected over a 2θ range of 35–135° with a step size of 0.01°. The lattice parameter of the phases was determined from the Rietveld method to attain a high degree of precision^25^.

### Advanced AFM techniques

As noted above, the main aim of this work is to determine the mechanical properties of nanostructured steels at the nanoscale in order to be able to assess whether both phases have different Young’s modulus or not. Accordingly, the most suitable technique, in principle, to address such investigation is the recently introduced atomic force microscopy mode, i.e., peak-force quantitative nano-mechanical, PF-QNM, imaging, which allows to map the local elastic properties with lateral nanometer resolution^26^,^27^. This method is based on the acquisition of force curves recorded at each pixel of the topographic image. The force curves are analyzed instantaneously. Then, provided that parameters such as cantilever spring constant and tip geometry are calibrated, the quantification of the nano-mechanical properties is possible. In the present configuration this analysis is done through the Derjaguin–Müller–Toporov (DMT) fit model of the retracing curve^28^,^29^ for a spherical indenter, which accounts for the adhesion effects between the sample surface and the tip, according to the equation:


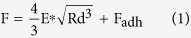


Thus, F is the applied force, R is the tip radius, d is the deformation value at a given force, F_adh_ the maximum adhesion force, and E^*^ is known as the effective elastic modulus, which is defined as:


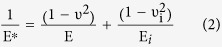


With E (ν) and E_i_ (ν_i_) being the Young’s modulus (Poisson’s ratio) of the sample and indenter, respectively.

As these calculations are done in real time for every force-distance curve obtained at every imaging pixel, the topography and nano-mechanical properties can be sampled simultaneously. Moreover, the latter data are also displayed as top-view image just as the topographical one (the z-axis being the Young’s modulus, E). This method then allows to measure the local nano-mechanical properties with the same lateral resolution than in the topographical image. This fact turns it as very suitable technique to assess these properties in the nanostructured bainitic steels. However, it is worth noting that this technique has been mostly employed to study the nano-mechanical properties of soft surfaces such as polymers^30^,^31^, biological entities as fibrils^32^, living cells^29^,^33^ and even nano-bubbles^34^. In contrast, scarce studies have been devoted to sample the nano-mechanical properties of hard surfaces. Thus, PF-QNM has been applied to study the Young’s modulus of WC/a-C coatings, in the 100–200 GPa range^35^, nano-structured Ge surfaces, in the 50–100 GPa range^36^, and hardened cement paste, up to 100 GPa^37^.

Specifically, quantitative mapping was performed at room temperature with a Multimode 8 (Veeco) and using a Nanoscope V controller (Bruker). Samples were imaged by using AFM operating in PF-QNM in air at a scan rate of 1.0–1.2 Hz. The AFM probe used in these studies was a diamond tip, model MDNISP-HS, (Veeco), with a resonant frequency of 63 kHz, spring constant of 402 N/m, and nominal radius of 45 nm. The loading forces during the measurements were kept close to 2 μN range. The diamond tip was calibrated initially on sapphire surfaces in order to set the deflection sensitivity. Once calibrated, the tip was used for PF-QNM imaging. It should be noted that the laser spot was kept at the same location on the cantilever during all the measurements. Finally, it should be commented that the diamond tip can eventually become contaminated or dirty during the QNM measurements. When this occurs, the tip is cleaned by performing indentations on a gold surface.

For the second set of experiments, i.e., the nanoindentation analysis, a Nanoscope IIIa (Veeco) equipment operating in tapping mode was employed. In this case, a diamond probe (DNISP, Veeco) with a resonant frequency of 64 kHz, nominal radius of curvature of 45 nm, and a spring constant of 247 N/m was used. The same calibration and cleaning procedures as those described in the QNM experiences were followed also in this case. Now, the measurement procedure was as follows: First, a tapping image was obtained and then an array of indentations was performed by changing the lateral position of the tip over the surface. At each spot of the array a single force curve, at a rate of 3.5 Hz and controlling the maximum load force (the highest value applied was of 200 μN), was done. In a final step, the tip was changed for a silicon one, with a nominal radius of curvature of 8 nm, in order to locate the area in which the indentation array was made and then measure it in contact mode in order to have more reliable information on the indentation geometry (i.e., area and depth) since the diamond tip is too wide. The analysis of the force curves (see below) in terms of the quantification of the Young’s modulus is done at the initial stages of the loading curve, i.e. at the elastic regime, by applying the Hertz model, in which adhesion effects are not accounted for, for different indenter geometries^38^. Specifically, we have fitted the loading force curves for spherical, conical and berkovitch indenter geometries, and we have found that the one leading to the best fittings was the spherical one. This fact allows us to better compare the analysis of the single indentations with the data obtained by PF-QNM since both analyses are done for the same tip geometry.

For this geometry, the relationship between the applied force, F, and the indentation, δ, is:


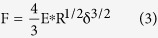


where R is the indenter radius and E^*^ is known as the effective elastic modulus, which was defined in eq. 2.

Provided that the cantilever has been calibrated on a hard surface (sapphire), its force constant and R values are known, the curve F = F(δ) can be obtained and then from a logarithmic plot the value of E can be derived. At this point, it is worth to comment on the errors involved in these measurements. The main source of errors is the value of R, which is quite difficult to know exactly (as well as the exact indenter shape at the very end of the tip). However, in this work we are mostly interested in detecting differences between the E values on the different phases present in the nanostructured steel than in determining their exact values.

**Figure 1:**
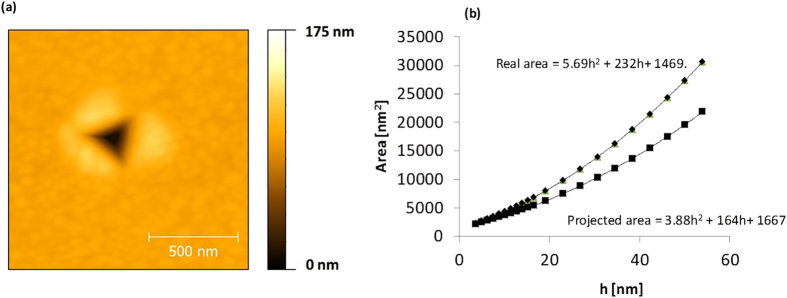
Topographical map of the surface of a gold sample, scanned with a fine tip, and showing the nanoindentation footprint made by a diamond tip (**a**), with its corresponding area-depth relationship (**b**).

The geometry of the tip was measured following this procedure: A sample of gold, considered as a perfectly plastic metal, was nanoindented with the diamond tip, and the footprints were then scanned using a finer tapping tip, Fig. 1a. A relation between depth and area of the tip was obtained, plotted in Fig. 1b. The depth, h, is measured with respect to the lowest height value of the topographical map scanned over the indentation. The projected area is the area of the footprint section parallel to the view’s plane, at every depth value, whereas the real area accounts for the whole surface below, i.e., the gold surface in contact with the tip. Besides the target samples, HT250 and HT350, fused silica was used as a reference sample, nanoindented with AFM in order to rule out possible artifacts in force curves not related to the material mechanical behavior.

**Figure 2:**
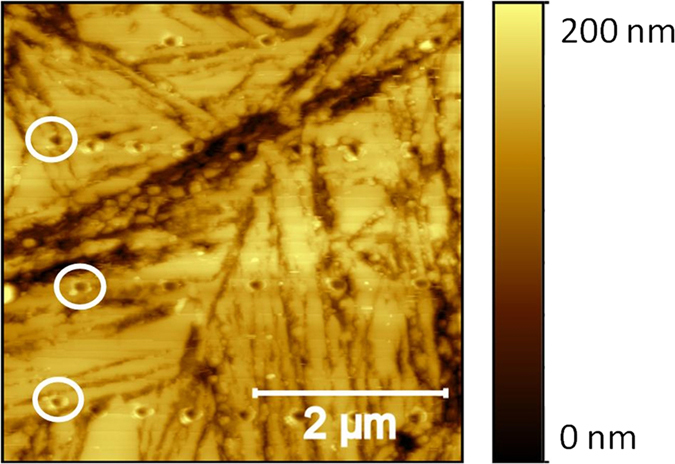
Topographical map of the etched surface of HT350 sample. The elevated area (brighter) corresponds to austenite, while the depressed (darker), to bainitic ferrite. Some of the nanoindentation footprints of the array are indicated with white circumferences for the sake of clarity.

A typical array, performed on HT350, of seven rows each containing eleven nanoindentations, as measured in contact mode, is displayed in Fig. 2. It is observed that the indentation rows present in some cases some sort of distortion regarding their lateral position. This is due to the lateral drift of the piezoelectric. The holes are mostly ∼1 μm apart from each other. At each point the corresponding force curve has been recorded and analyzed in order to obtain the E value (see below).

## Results

### Initial microstructure

**Figure 3:**
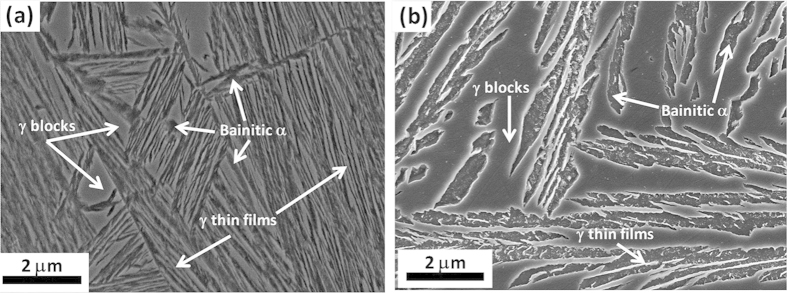
Secondary Electron SEM micrograph of HT250 sample (**a**) and HT350 (**b**). Non-etched regions correspond to austenite (γ) which displays two different morphologies, block and thin films, whereas etched regions correspond to bainitic ferrite (α).

**Figure 4:**
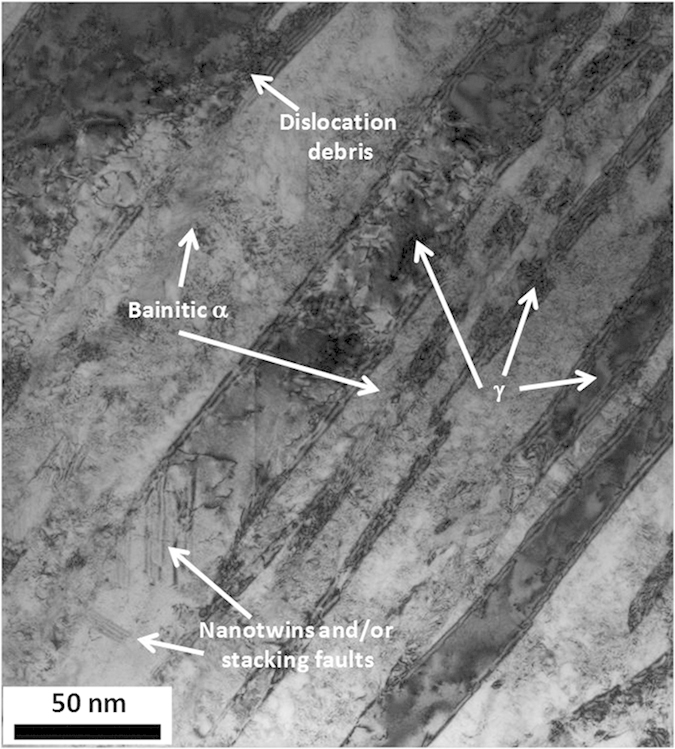
TEM micrograph of sample HT250, detailing the presence of austenite (γ) and bainitic ferrite (α), and defects.

Nanostructured bainite is formed by a body-centered cubic (bcc) nanoscale matrix (ferrite) and a face-centered cubic (fcc) second phase (austenite). Both phases can be observed in the secondary-electron SEM image, Fig. 3, of etched surface samples. Austenite (γ), which is the dispersed second phase, has two different morphologies namely, thin films between the plates of ferrite (α), both in the nanoscale range, and coarse blocks, up to the submicron scale. From Fig. 3, it is evident the effect of the heat treatment temperature on the scale of the microstructure: The sample treated at higher temperature, HT350, exhibits much coarser microstructural features than HT250. Microstructural differences between both samples go beyond those observed by SEM, and higher magnification techniques are required to detect the complexity and nanoscale nature of these structures. Bainite reaction occurs via a displacive and diffusionless, solid-solid phase transformation, where there is no change in the chemical composition between the parent and product phase. Transformation is accompanied by plastic relaxation of the shape change occurring as a consequence of the mentioned displacive growth of bainite, which takes places via generation of both, dislocations in the austenite/bainitic ferrite interface as well as via micro/nano twins in the austenite in contact with bainitic ferrite. This plastic relaxation produces the appearance of inhomogeneus residual microstrains. The TEM image of HT250 in Fig. 4 shows bainitic ferrite plates (brighter regions) between films of retained austenite (darker regions), exhibiting evidences of dislocation debris and other defects as nanotwins^39^,^40^,^41^. Once the diffusionless growth of a bainitic ferrite subunit has ended, the excess of carbon partitions into the surrounded austenite. The process continues by successive nucleation of subunits until the carbon concentration of the residual austenite reaches the value at which the free energy of ferrite becomes less than that of austenite of the same composition; the transformation stops at that point because it is thermodynamically impossible for the transformation to proceed by a diffusionless reaction^42^. Carbon enrichment makes the austenite thermally stable, avoiding martensitic transformation upon cooling at room temperature. Carbon lies not only at defect free solid solution, but also at the huge amount of dislocations, boundaries, and clusters present. However, during bainitic reaction, massive carbide precipitation is avoided thanks to the use of Si as alloying addition. Therefore, the two final phases are the depicted bainitic ferrite and retained austenite, whose lattice parameters are listed in Table 1. As it has been recently proven, bainitic ferrite is tetragonal rather than cubic, increasing its carbon solubility^43^,^44^,^45^.

### Elastic properties

Loading force curves recorded during the nanoindentations, are analyzed at their very beginning, i.e., within the elastic regime. The method consists in fitting the first stage of the curve to the Hertzian solution of Eq. (1) so that E can be obtained, assuming that R = 45 nm; K = 247 N/m; ν = 0.3.

The nanoindentation process was first performed on a homogeneous and isotropic sample, fused silica, used as a reference, with a known E of 75 GPa and a Poisson’s ratio, ν, of 0.17. The force curves analysis resulted in an average E value of 75 ± 15 GPa. The obtained error range should be, in principle, mostly attributed to the intrinsic limitations of the technique.

**Figure 5:**
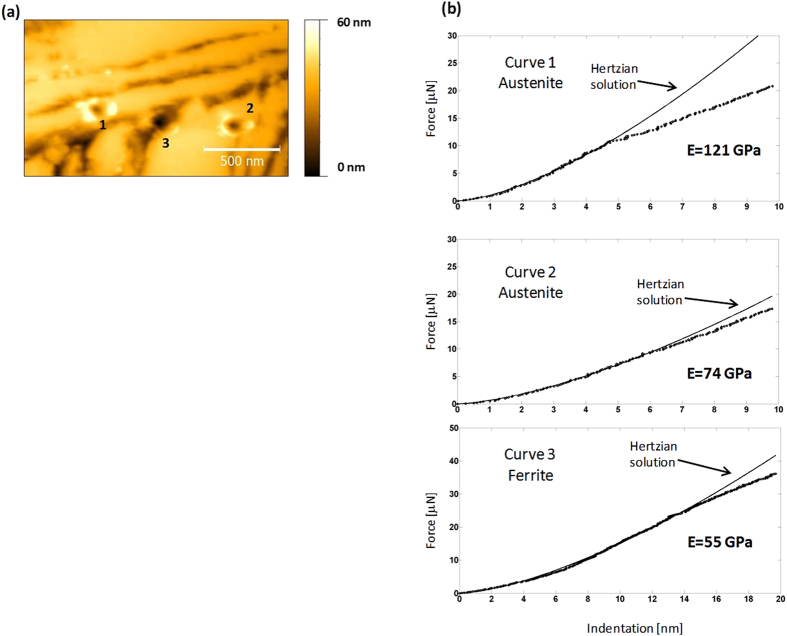
Details of topographical maps of the etched surface of HT250 with three numbered nanoindentations produced by AFM (**a**); and their corresponding force curves (**b**).

The same procedure was followed for sample HT250. The aim of this study was to be able to identify the phase nature of each spot in which an indentation had been performed. In principle, this would be possible because the etching process with Nital would have revealed both phases, i.e., the austenite lying at more elevated locations than the ferrite, the latter being preferably etched. However, this task proved to be rather difficult due to the nanostructured nature of the steel. Thus, most nanoindentation footprints in HT250 were found over more than one phase or at grain boundaries turning the corresponding phase identification quite ambiguous. An example is shown in Fig. 5a where it is observed that even those indentations performed on the austenite phase, i.e. the smooth plateau-like regions, are close to, or even affect to, etched zones. This problem also is evident in the central indentation that was made on a presumed ferrite nanodomain. In this case, it is also clear that the indentation has been made on a locally rough or stepped surface. Thus, it should be stressed that it was statistically quite improbable to indent unambiguously in a single phase.

**Figure 6:**
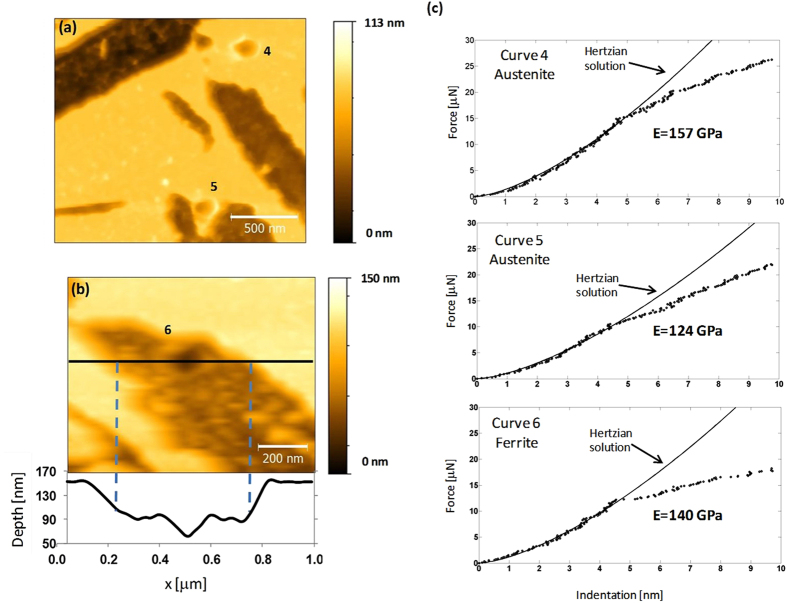
Details of topographical maps of the etched surface of HT350 in which footprints of nanoindentations are numbered (**a**) and (**b**); and their corresponding force curves (**c**). The nanoindentation footprint 6 (**b**), in ferrite, is presented together with its surface profile along the horizontal direction.

In Fig. 5b the corresponding force curves, plotted as force versus indentation, are displayed in which the Herztian regime is shown. Also, it is worth noting that at the end of this regime there is a clear crossover to a linear regime, which is related to the yielding process (see below). However, when we analyzed the Hertzian regime, we obtained E values that were appreciably lower than the expected ones. This fact could be due to the relatively jagged morphology on which most of the indentations were performed as a consequence of the etching of the nanostructured bainite. In order to overcome this problem and to try to measure reliably the E value of both phases, we followed the strategy of studying the same steel treated at a higher temperature, in order to obtain a coarser bainite structure (sample HT350). The goal of this approach was to perform unambiguously the indentations on well-defined phases. Typical examples of these experiments are shown in Fig. 6a,b. Now, nanoindentation footprints can be seen over clearly identified phases. However, only in the case of austenite, it has been possible to identify unambiguously the phase in a high number of nanoindentation footprints. The corresponding force curves are shown in Fig. 6c. There is a good fitting between the theoretical Hertzian curve and the experimental data up to the critical point where plastic regime starts, at which both curves diverge. The onset of the plastic regime and its evolution will be discussed later, after elastic properties had been evaluated.

**Figure 7:**
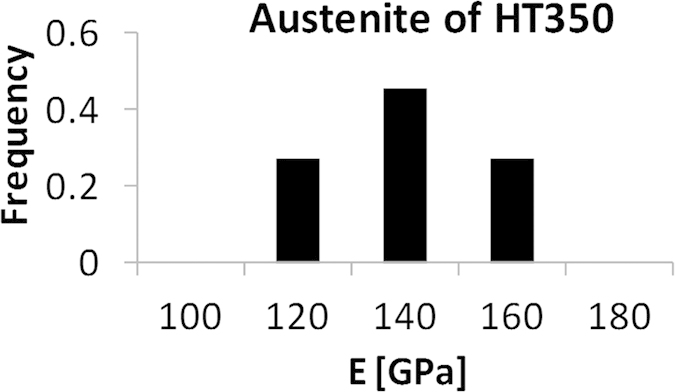
Normalized distribution of E values obtained by AFM nanoindentations in austenite of HT350.

From Fig. 5, E values of bainitic ferrite in HT250 result to be extremely low, compared to those reported in literature where for most steels E has a value of 200 ± 15 GPa^46^. These results can be due to the finer scale of the HT250 microstructure, implying more artifacts caused by the etched-induced topography. If we restrict our study to the case of the austenite phase which is coarser and flat, the analysis of the whole nanoindentation arrays revealed a wide spread of values for HT250 austenite: 50 GPa < E < 200 GPa. In contrast, for austenite of HT350, there were more analyzable curves, enough to build a reliable normalized E distribution, Fig. 7, with E values of austenite, between 120 and 160 GPa, i.e., slightly lower than the macroscopic E value, about 180 GPa. The spread in the measured E values may come from different sources. As explained, an approx. error of 20% is inherent to the technique. But differences in E values may also arise from the complexity of the bainitic microstructures, comprising two different and heterogeneous phases, each consisting of features with different crystallographic orientations and also a heterogeneous carbon distribution in both solid solution and located at defects. In fact, when considering only the elastic anisotropy, E has been reported to vary in a range of approx. 50 GPa depending on the plane family considered, by diffraction techniques, in similar microstructures^47^. However, the fact that the E values obtained for the austenite phase on HT350 are higher, and closer to the expected ones, than those measured on HT250, suggests that other issues can come into play such as the surface topography, not only the roughness but also the local morphology. Furthermore, the AFM indentation array experiments can also imply additional problems or limitations such as the eventual contamination of the tip as relatively high loads (in the AFM range) are applied. Besides, the discreet sampling, the complexity of the bainitic microstructures as well as the blind character of the experiment hamper obtaining reliable statistical data on both phases, particularly on the ferrite one.

Thus, at this stage of our work, we decided to address this study by means of PF-QNM. This technique allows to measure the topography and E value of the surface simultaneously and continuously, i.e. on the whole imaged area. Furthermore, we have operated the microscope using extremely low loads, at 2 μN, which is considerably lower than those used in the indentation experiments. This fact reduces the probability of tip degradation and contamination. In any case, once the tip becomes contaminated there is a sharp reduction in the E values that allows us to stop the measurement and to proceed to clean the tip by indenting the gold surface as commented in the experimental section. This is a sort of *in-situ* checking of the tip status.

**Figure 8:**
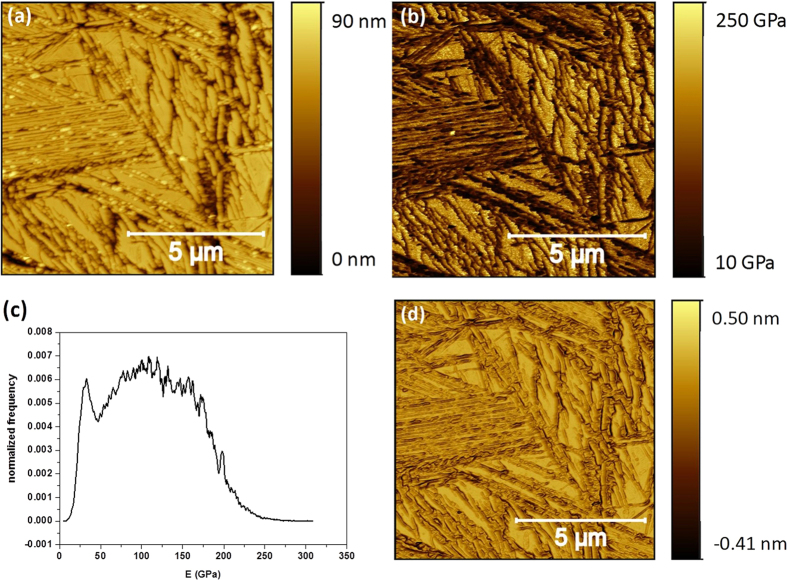
The topographical map (**a**) and E map (**b**) of sample HT250 with etched surface, measured by PF-QNM; together with its normalized E distribution (**c**) and the corresponding image of the inverse of the slope of Fig. 8a (**d**). The inverse of the slope has been obtained in order to follow the same scanning sense, from right to left, of the tip along the horizontal axis (**d**).

First, we analyzed the HT250 sample. In Fig. 8a,b are displayed the topographical and E maps measured simultaneously by PF-QNM. The topography shows the jagged morphology commented above with several crevices and deep grooves produced as a consequence of the preferential etching of the ferrite phase. This morphology results in an average roughness of 12 nm. In Fig. 8b the corresponding E map is displayed in which the austenite plateau-like phase appears to have a high E, in the 130–190 GPa range, whereas dark patches with extremely low E values, close to 20 GPa in some cases, are also measured. The E image leads to a normalized E distribution plot as that depicted in Fig. 8c, with a sharp peak centered at 30 GPa and a broader one within the 60–220 GPa range. A careful inspection of Fig. 8a,b shows that the low E patches are clearly related to the etched zones. Therefore, the straightforward conclusion would be to associate them to the ferrite phase. However, the extremely low E values reached at some spots suggest that a more detailed analysis is required. Thus, we decided to obtain the slope image corresponding to Fig. 8a. This is shown in Fig. 8d (see caption). From the comparison of images Fig. 8b,d, it becomes evident that there is a direct correlation between the slope of the morphology and the obtained (low) E values. This was confirmed by performing cross-sections of both images along the same paths (not shown). Therefore, low E values were obtained when sharp discontinuities or slopes were present in the surface morphology. This fact prevents us to associate the low E values to the ferrite phase. In contrast, as the austenite phase has a smooth and flat morphology the E value measured on these regions, 130–190 GPa, is more reliable. However, even in this case some influence of the morphology cannot be discarded as the flat austenite plateaus still display a certain roughness at the nanoscale level.

**Figure 9:**
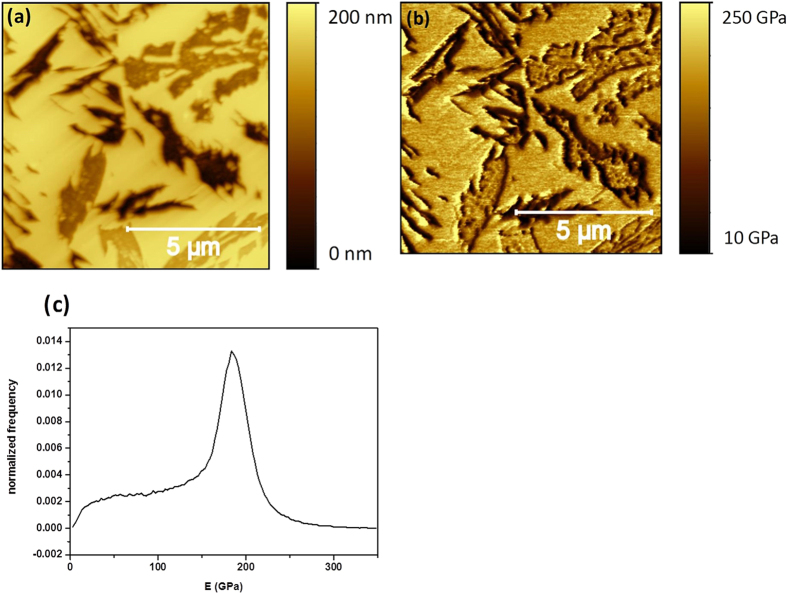
The topographical map (**a**) and E map (**b**) of sample HT350 with etched surface, measured by PF-QNM; together with its normalized E distribution (**c**).

In order to overcome this limitation imposed by the etching induced topography on the PF-QNM measurements of sample HT250, we proceeded to apply this procedure on the HT350 sample. In this sample, we expected that the etching would lead to ferrite etched phases wide enough to be rid of topographical contamination on the determination of the corresponding E values. Figure 9a,b show characteristic topographical and E maps, taken simultaneously in the PF-QNM mode, on the HT350 system. In the topographical images the austenite domains are clearly visible as they correspond to the higher (brighter) locations that display a flat plateau-like morphology, with a roughness of 1.5 nm. In addition, deep crevices, as deep as 100 nm, have been produced by the etching procedure, leading to a general surface roughness close to 38 nm. In some cases, the bottom of these deep crevices is quite flat and wide (i.e. larger than 100 nm). This is more evident for other shallower crevices or etched structures that are around 20 nm deep (i.e., at the right bottom and top parts of the image, for instance). In these cases, the bottom regions are also at least 100 nm wide and flat (i.e. with a roughness of 3 nm). Thus, in principle we should not expect strong artifacts coming from the morphology at these bottom crevices of the ferrite phase. This is confirmed when we analyze the corresponding E map (Fig. 9b). Once more, the sharp perimeters of the crevices display a dark contrast, i.e. low E values, in agreement with the observations realized in sample HT250. However, this time most of the crevice bottoms present a higher (i.e., larger E values) and homogeneous contrast. In fact, the corresponding normalized E distribution (Fig. 9c) shows now a clear peak at 185 GPa with a long tail down to the low E value region. This tail is due to the dark E regions corresponding to large sloped morphological regions. A careful analysis of the wider ferrite exposed domains reveals a slightly lower E value than that found on the higher flat austenite domains. More specifically, these ferrite zones show an E value close to 165 GPa whereas the austenite ones present values in the 185 GPa range.

Therefore, by revealing the ferrite and austenite phases on a sample with wider domains, we have been able to measure the E value of each phase under the same conditions, and assess that they are quite similar within the error of the PF-QNM measurement mode. One final way to confirm this result is to measure by the same technique the polished samples without further etching. In this way, on one hand, we will deal with flat surfaces. Therefore, the measurements will be free from strong morphological induced artifacts as those discussed above for the etched samples. On the other hand, we will not be able to identify each phase.

**Figure 10:**
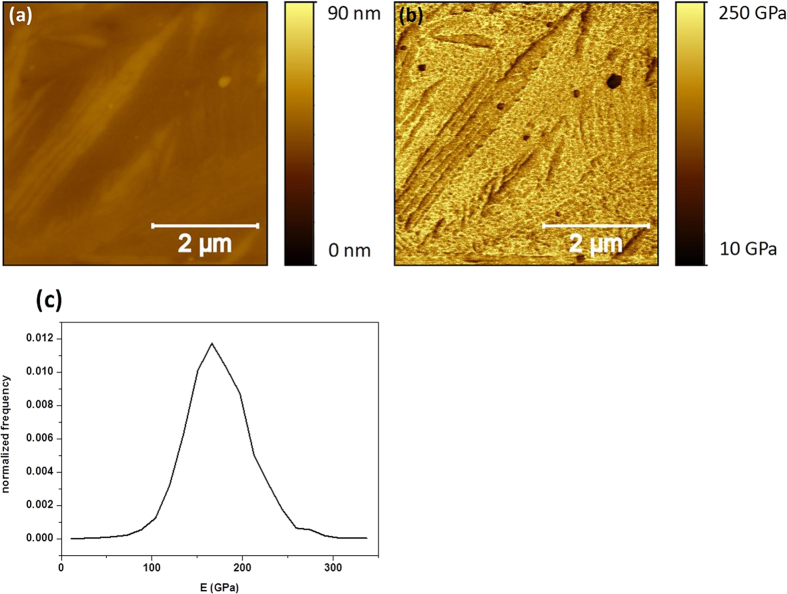
The topographical map (**a**) and E map (**b**) of sample HT250 with polished surface, measured by PF-QNM; together with its normalized E distribution (**c**).

**Figure 11:**
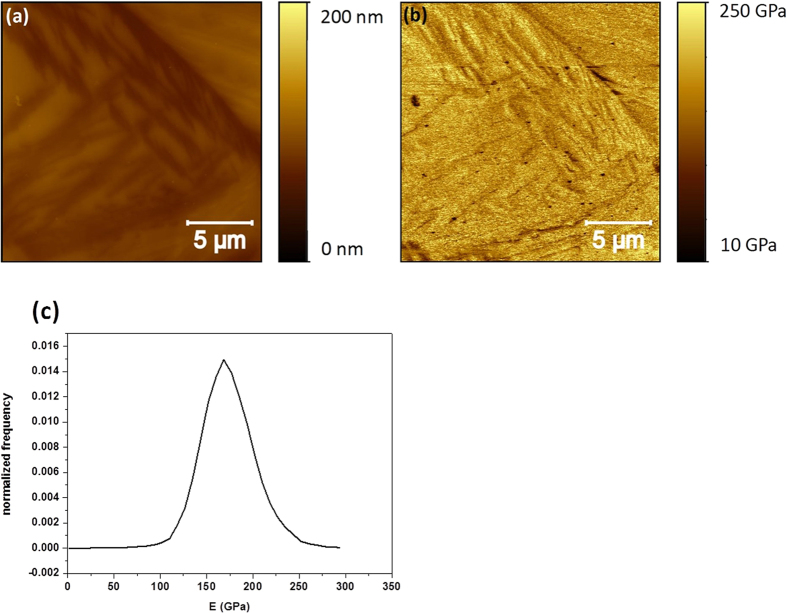
The topographical map (**a**) and E map (**b**) of sample HT350 with polished surface, measured by PF-QNM; together with its normalized E distribution (**c**).

In Fig. 10a,b are displayed the topographical and E maps of the HT250 polished sample. Clearly, the surface is much smoother, with a roughness below 1 nm, than in the etched ones. Still, some structures are visible with heights in the 10 nm range. The corresponding E map, in contrast, is quite homogeneous, although it can be noticed that the steps of the above mentioned structures still give lower E values, in agreement with previous results. However, the average E value results to be 184 ± 35 GPa. The corresponding normalized E distribution is plotted in Fig. 10c. The corresponding data for the HT350 polished sample are shown in Fig. 11a–c. Again, the surface still presents some morphological features with height differences close to 10 nm, but a surface roughness below 1 nm (Fig. 11a). The corresponding E map is, in this case, quite homogeneous (Fig. 11b) with a relatively narrow E distribution (Fig. 11c). The average E value is 178 ± 30 GPa. The E values obtained in both polished samples are quite consistent with those measured on the austenite and ferrite phases in the corresponding etched samples. The fact that the E maps of the polished samples are quite homogeneous implies that both phases, indeed present at the surface, do have similar E values, in agreement with our results obtained on the etched samples by PF-QNM and on etched HT350 sample by nanoindentation.

## Discussion

The results evidence the advantages and limits of performing nanomechanical measurements with the AFM based modes employed in this work. Regarding the E analysis from AFM indentation curves, it is clear that their interpretation is hampered both by the sample morphology and by the experimental procedure itself. In the first case, as we have seen also in the PF-QNM case, the surface roughness and the ambiguity in performing the curves on well-defined nanophases are the main source of errors. These two effects usually lead to E values smaller than expected, just as it occurs in PF-QNM experiments but even to a larger extent. In addition, the problems coming from eventual contamination of the tip during the indentation array experiments can also result in large deviations in the experimental results.

However, despite these limitations and problems, the AFM indentation data can still be further analyzed in other ways. As noted previously the crossover point at which the Hertzian behavior fails is known as the yielding point. It is related to the onset of the plastic behavior of the given domain. Therefore, we can investigate whether the corresponding yielding force depends on the nature of the domain. This information could be valuable due to the complexity of the microstructure.

Thus, the stress at which each phase yields can be estimated directly from the nanoindentation force curves, and for this purpose the corresponding E values will be used. It is known that the maximum shear stress (τ_max_) of the elastic regime in a nanoindentation reads as equation 4^48^:


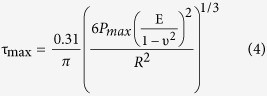


where P_max_ is the maximum load applied at the elastic regime, i.e., the maximum deflection multiplied by K, the spring constant. Obviously, after the previous analysis on Young’s modulus, it is reasonable to apply eq. 3 only to curves which seem to be not affected by topography, i.e., curves 4, 5 and 6 of HT350, for which calculated E values are more reliable. The force value at which yielding starts is assumed to be the point at which force curve presents an abrupt change of trend, getting away from the elastic theoretical solution. P_max_ and τ_max_ are shown in Table 2.

These τ_max_ values are far above the shear stress at which macroscopic plastic deformation is measured (1/2(Yield Strength)), but in good agreement with results obtained for other steels^49^. Data of Table 2 suggest that yielding might need a similar or even lower shear stress to occur in bainitic ferrite than in austenite belonging to HT350. These results are opposed to what other authors have reported for nanostructured bainite, in which bainitic ferrite is believed to be the hardest phase^50^, as also seems to occur for other microstructure, like Q&P steels^6^.

An interesting feature observed in the load-displacement curves displayed in Fig. 6, is the appearance of discontinuities or local changes of slope once the observed behavior deviates from the Hertzian solution. The first slope change is the most marked and corresponds to the onset of the plastic behavior, just at the point where the load curve deviates from the Hertzian fit whereas for further displacement values, less marked slope changes can be observed. These features, akin to those observed during nanoindentation tests, are related to the occurrence of certain deformation mechanisms, and imply, for instance, displacement bursts^49^. Among the mechanisms responsible of such discontinuities in the curves, we can mention the load-induced dislocation nucleation and the sudden increase of mobile dislocations^51^. This is the case of the ferrite phase where the source of such effects could be the unpinning of dislocations which were previously locked by Cottrell atmospheres, i.e., C trapped at the dislocations^52^. The presence of these Cottrell atmospheres is common in bainitic ferrite, as extensive Atom Probe Tomography examination has revealed^53^. For the austenite phase, however, these events are usually related to the mechanically-induced martensitic transformation of austenite, triggered by the plastic deformation^49^,^51^,^54^. Finally, other possible mechanism that could also explain these features is the surface roughness^55^.

**Figure 12:**
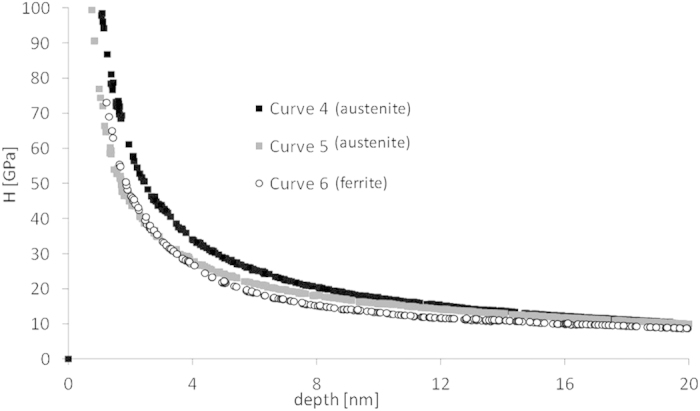
Evolution of H with indented depth for nanoindentations 4, 5 and 6 in sample HT350.

To complement this analysis, it is worth assessing the evolution of local values of hardness corresponding to nano-indentations, 4, 5 and 6 of HT350, Fig. 12. Meyer hardness (H) is defined as P (force applied) over the projected area of the indentation for that P value. In this work, the projected area, for each P value of the force curve, is approximated according to equation of Fig. 1b, where depth is now the “plastic” indentation: the corresponding indentation, δ, minus the indentation at the yielding start of the force curve. The gradual decrease of H as a function of depth is due to the size-effect of the indenter^56^. Thus, it is advisable to compare values of H for a same value of depth. For a depth above 4 nm, the H value of curve 6 (bainitic ferrite) is lower than the others, curve 4 and curve 5 (austenite), which can be associated to a higher availability of austenite to work-harden. By comparing curves 4 and 5 (austenite), it is noticeable that their H values converge at higher values of depth, when the effect of different yield stresses dissipates.

Regarding the results obtained through the PF-QNM technique, it is evident that the capacity to directly correlate the topographical and E maps, contribute to identify those data where the topography does contaminate the E evaluation. Thanks to this simultaneous sampling we have been able to perform reliable imaging of E values on the austenite and ferrite nanophases. In fact (see Fig. 9) we have detected that ferrite domains displayed slightly lower E values, around 15–20 GPa smaller than austenite ones sampled under the same conditions. However, the consideration of the normalized E distributions for the polished samples, leads to the conclusion that errors involved in the measurements can be of this order of magnitude. It should be noted that in even these polished samples the nano-roughness of the mostly flat surface can also play a role in the distribution of the E measured values. Thus, although this slight difference may be observed in Fig. 9, we cannot state unambiguously that there are such differences.

Young’s modulus depends in an important part on the crystal structure and on interatomic distances. For a same structure, the higher the lattice parameter, the lower the Young’s modulus^57^,^58^. In some ultrafined steels, the refinement of the microstructure is linked to a decrease of the lattice parameter and the corresponding increase of Young’s modulus, even in more than 20% for a lattice parameter difference of 0.005 Å^57^. In carbon steels, the lattice parameter is highly dependent on the carbon content in solid solution, as it is normally located at interstitial sites. In those steels, different carbon contents can result in Young’s modulus differing in up to 10%, according to macroscopic mechanical tests^46^. Comparing phases, ferrite has in theory a slightly but not negligible higher Young’s modulus than austenite. However, as ferrite turns from a cubic structure into a tetragonal one, with the corresponding increase of carbon content, the Young’s modulus has been reported to decrease down to approx. 5%, both phases tending to equal their values^8^,^56^. It is important to note that if ferrite and martensite have a different chemical composition, apart from the carbon content, that trend is not the necessarily true, i.e., ferrite may have a lower Young’s modulus than martensite, as observed by nanoindentation in Q&P steels^6^. In bainitic steels, austenite and bainitic ferrite only differ in their carbon content, the rest of the alloying content being the same. In the case of bainitic ferrite, there are recent proofs confirming that the tetragonality of the body centered structure is directly proportional to the carbon content in solid solution, in the same manner as martensite^43^,^44^,^45^. Different bainitic heat treatments result in differences in carbon content of the phases, affecting their lattice parameter, and, in turn, the Young’s modulus. Apart from the average lattice parameter, inhomogenous microstrains and carbon distribution may also cause the spread of local Young’s modulus values.

Austenite from HT250 and from HT350 differ in their average lattice parameter in 0.004 Å due to the differences in their carbon content, which is the only element partitioning from bainitic ferrite into austenite after transformation. However, the quantitative resolution level is not good enough to assess any difference between the E values of the austenite phase in the HT250 and HT350 samples below 10%.

Despite the limitations of PFT-QNM to reveal unambiguously the differences below a certain level of the contributions of the different phases to the overall E values, the main advantage of the technique is that it can assess whether there are clear differences in the elastic response of both phases coexisting at the steel surface, when both have large enough lateral dimensions as in HT350. In this sense, it is necessary to remark that the measurements take place under the same conditions, particularly those referring to the tip status. Furthermore, as they are performed at so low loads, close to 2 μN, we can discard any effect on the observed behavior of each sampled phase coming from the surrounding, either laterally or along the vertical direction, phases. Experimental results do allow us to state that both phases have quite similar Young’s modulus within the experimental error bar. Thus, in terms of Young’s modulus the steel would be a quite homogeneous material despite its nanostructured nature.

## Conclusions

In this work we have addressed the evaluation of the Young’s modulus (E) of the austenite and ferrite nanophases forming the nanostructured bainitic steel. A special emphasis has been addressed to discern whether they present a clear difference in Young’s modulus. For that purpose we have applied two AFM-based techniques namely, AFM indentation and PF-QNM. The former allows to perform very shallow and small indentations whereas the later allows to map the sample stiffness with high lateral resolution and simultaneously to the conventional AFM morphological imaging. Evaluation of the E values of the different nano-phases by AFM indentation experiments was affected by different limitations and problems. In contrast, these experiments were also analysed in terms of the phase yielding behaviour. In this sense, the critical shear stress necessary for yielding initiation seems to be equal or even lower for bainitic ferrite than for austenite, in the sample treated at higher temperature, 350 °C.

Due to the problems found for the evaluation of the E values of each phase by AFM indentation, we performed further experiences by PF-QNM. However, studies carried out on the nanostructured HT250 showed that the AFM-based measurements of E were largely affected by the etched morphology. Thus, it was only possible to obtain reliable data on the flat austenite phase. In order to be able to measure the E value of both phases in the same experiment, we performed this analysis on the HT350 steel, which displayed larger domains but still in the submicrometer range. The PF-QNM studies allowed us to conclude that the observed differences in Young’s modulus between austenite and bainitic ferrite seem negligible, with E values close to 180 GPa. These results were also confirmed by measurements on the corresponding polished samples in which homogeneous E maps were obtained. This lack of heterogeneity implies a similar E value for both phases present at the polished surface, although undistinguishable topographically. Therefore, these results imply that these steels, despite their nanostructured compositions, are homogeneous in terms of Young’s modulus.

## Additional Information

**How to cite this article**: Morales-Rivas, L. *et al.* Nanomechanical characterization of nanostructured bainitic steel: Peak Force Microscopy and Nanoindentation with AFM. *Sci. Rep.* **5**, 17164; doi: 10.1038/srep17164 (2015).

## Figures and Tables

**Table 1:** Lattice parameters of austenite (a_γ_) and bainitic ferrite (a_α_ and c_α_).

Sample	a_γ_ [Å]	a_α_ [Å]	c_α_ [Å]
HT250	3.6190	2.8544	2.8792
HT350	3.6240	2.8545	2.8733

**Table 2:** Maximum force and the corresponding maximum shear stress of elastic regime in curves of Figure 6.

Sample	Curve	Phase indented	P_max_ [μN]	τ_max_ [GPa]
HT350	4	Austenite	11.51	10
	5	Austenite	11.02	8
	6	Ferrite	11.02	9
